# Measurement accuracy of dental cone-beam computed tomography for assessing submillimeter distances between implant and mandibular canal: a phantom study

**DOI:** 10.1186/s40729-026-00694-2

**Published:** 2026-06-07

**Authors:** Hozumi Yoshihara, Eiichi Honda, Yoshizo Matsuka

**Affiliations:** 1 Department of Oral and Maxillofacial Radiology, Tokushima University Graduate School of Biomedical Sciences, Tokushima, Japan; 2 Division of Quantum Science and Technology in Oral and Maxillofacial Regions, School of Dentistry, Aichi Gakuin University, Nagoya, Japan; 3 Department of Stomatognathic Function and Occlusal Reconstruction, Graduate School of Biomedical Sciences, Tokushima University, Tokushima, Japan

**Keywords:** Cone-beam CT, Dental implants, Mandibular canal, Titanium

## Abstract

**Purpose:**

Accurate assessment of the distance between dental implants and the mandibular canal is essential for preventing nerve injury. Although cone-beam computed tomography (CBCT) is widely used for implant planning, its accuracy in resolving submillimeter distances remains uncertain. This study evaluated the measurement accuracy of CBCT for assessing distances below 1.0 mm.

**Methods:**

A custom phantom enabling 0.0–1.0 mm implant–canal distances in 0.1-mm increments was developed. CBCT images were acquired at varying distances, positional shifts (X, Y, Z), and tube voltages. Five dentists involved in implant treatment measured the implant–canal distances using medical-grade and general-purpose monitors. Measurement error and contributing imaging factors were statistically analyzed.

**Results:**

CBCT did not reliably distinguish distances ≤ 0.4 mm, with the greatest instability observed at 0.3 mm (interquartile range = 0.121). Although a linear trend was observed from 0.1 to 0.4 mm, variability exceeded clinically acceptable limits. For distances ≥ 0.5 mm, reproducibility was high, but CBCT consistently underestimated the true gap by 0.2–0.3 mm. The central field of view produced the most stable measurements, whereas accuracy decreased with off-center positioning. Tube voltage and monitor type had minimal influence on measurement accuracy.

**Conclusions:**

CBCT cannot accurately identify implant–canal distances ≤ 0.4 mm, which may directly affect clinical risk assessment. Even for distances ≥ 0.5 mm, CBCT underestimates the true distance by 0.2–0.3 mm. These findings provide practical guidance for setting safe margins in implant planning and postoperative evaluation.

## Introduction

In dental implant practice, accurate three-dimensional identification of the mandibular canal is critical. In particular, the distance between the implant apex and the superior wall of the mandibular canal is a key prognostic factor for postoperative neurosensory disturbance. At present, this distance is commonly evaluated using dental cone-beam computed tomography (CBCT).

CBCT has become an indispensable imaging modality for implant planning because it provides high-resolution three-dimensional images of hard tissues with relatively low radiation exposure. Although voxel sizes of approximately 0.1 mm are considered to allow submillimeter distance evaluation [1], technical constraints limit the effective spatial resolution of current CBCT systems to approximately 2.0 lp/mm at 10% modulation transfer function (MTF), corresponding to a spatial resolution of about 0.25 mm [2]. Displacement from the rotational center of the system may further reduce visual accuracy by approximately 0.4 mm [3].

Regarding clinical measurement accuracy, linear measurements using CBCT are reported to be accurate to within 0.5 mm, and with some systems or under specific conditions, errors can be as small as 0.2 mm [4]. Nevertheless, measurement errors vary substantially depending on the device, acquisition parameters, and presence of metal artifacts. In particular, around dental implants, beam hardening and partial volume effects may cause discrepancies between image-based measurements and true anatomical distances [5].

Medical multi-detector row CT (MDCT), which enables reconstruction with isotropic voxels, has reportedly improved three-dimensional spatial resolution to approximately 0.3 mm [6]. Studies on MDCT have shown that although visual measurement errors between the alveolar crest and the superior wall of the mandibular canal are smaller than those observed with conventional CT, errors of approximately 0.7 mm still remain [7]. These findings suggest that distance measurement in the submillimeter range is inherently challenging, regardless of imaging modality.

In implant treatment, proximity between the implant apex and the superior wall of the mandibular canal of less than 1.0 mm has been associated with neurosensory disturbances [8]. However, few studies have specifically focused on neurosensory changes related to distances of 1.0 mm or less [9, 10]. Accordingly, a minimum safety distance of at least 1.5 mm has been clinically recommended [11]. Nevertheless, the extent of “image-based measurement error” underlying this safety margin, particularly in submillimeter ranges below 0.5 mm, has not been quantitatively clarified. Moreover, most previous studies were performed on dry human mandibles, in which unavoidable measurement errors of approximately 0.1 mm arise from sectioning procedures and caliper measurements, potentially hindering the rigorous definition of true values [12, 13].

Furthermore, in CBCT imaging, small errors on the order of one voxel (approximately 0.1–0.35 mm) may be amplified when measurement distance increases, resulting in positional deviations of 0.2–1.5 mm at implant sites [14]. Therefore, to evaluate the boundary region of 0.2–0.4 mm, which is reportedly influenced by the outer margin of metal artifacts, an experimental system that can control distances in 0.1-mm increments is essential. To date, no studies have examined measurement accuracy while systematically varying distances of 1.0 mm or less under conditions simulating the mandibular canal and dental implants.

Accordingly, in the present study, we designed and fabricated a novel phantom simulating the mandibular canal and a dental implant within the mandible, with the distance between the two structures being adjustable in submillimeter increments. Using this phantom, CBCT images were acquired with varying implant–canal distances and acquisition parameters, and the distance between the implant apex and the superior wall of the mandibular canal was measured as in routine clinical practice. This study aimed to quantitatively evaluate the accuracy of CBCT-based distance measurements in submillimeter ranges (≤ 1.0 mm) and to clarify the limitations and clinical implications of assessing minimal implant–canal distances in dental implant practice.

## Materials and methods

### Phantom

In this study, a custom phantom with titanium screws and polytetrafluoroethylene (PTFE) tubes was used to simulate the implant body and the mandibular canal, respectively. Pan-head machine screws made of commercially pure titanium (manufacturer’s specification: Ti ≥ 99.6%; Trusco Nakayama Corp., Tokyo, Japan), compliant with ISO 261:1998, were used. The screw size was set to M3 (diameter, 3 mm; pitch, 0.5 mm; length, 10 mm), based on the approval standards for dental implants issued by the Ministry of Health, Labour and Welfare of Japan [15]. The screw tip was cut and polished with a carborundum point to obtain a flat central portion. Naflon® PTFE tubes (NICHIAS Corp., Tokyo, Japan) with an outer diameter of 4 mm and an inner diameter of 2 mm were used. The tube lumen was filled with a UV-curable acrylic resin (Padico Co., Ltd., Tokyo, Japan). Resin, utility wax, paraffin wax, and cyanoacrylate adhesive were used for fixing and bonding.

**Fig. 1:**
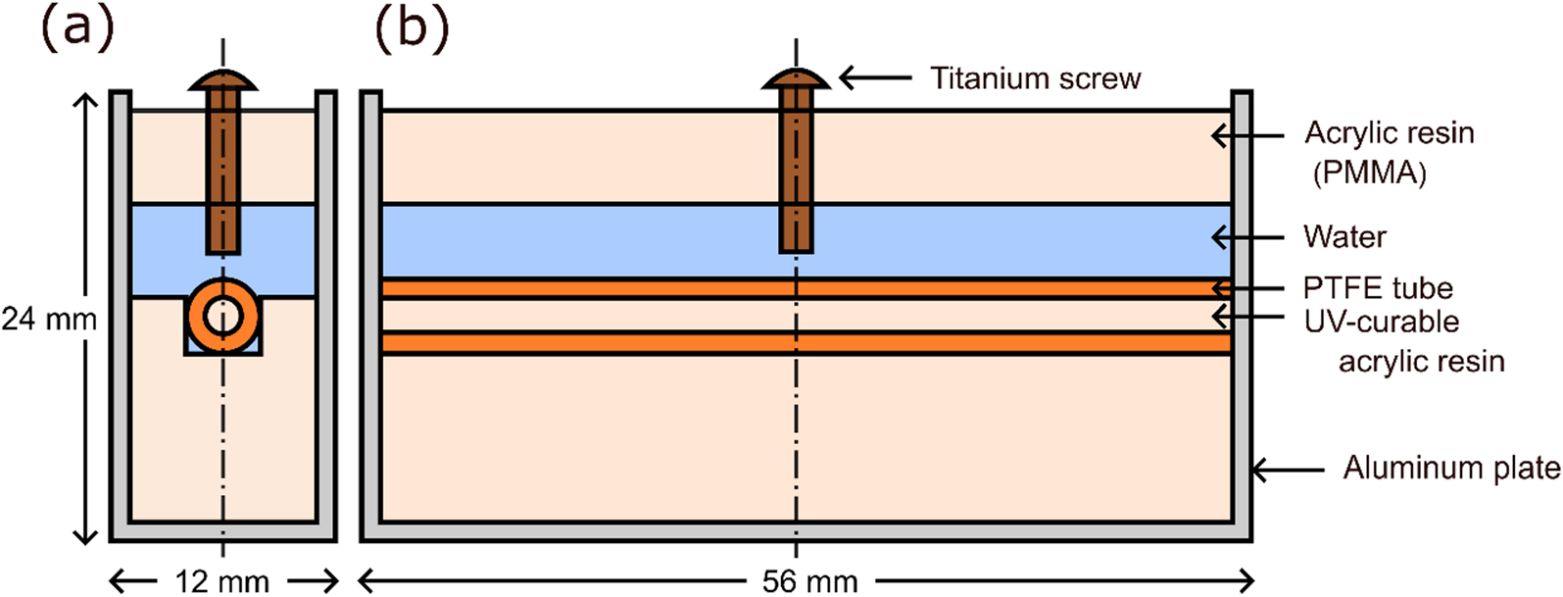
Schematic illustration of the experimental phantom. **a** Coronal view. **b** Sagittal view. The phantom consisted of acrylic resin (PMMA), water, a PTFE tube, UV-curable acrylic resin, and a 0.5-mm aluminum plate. PMMA: polymethyl methacrylate; PTFE: polytetrafluoroethylene

**Fig. 2:**
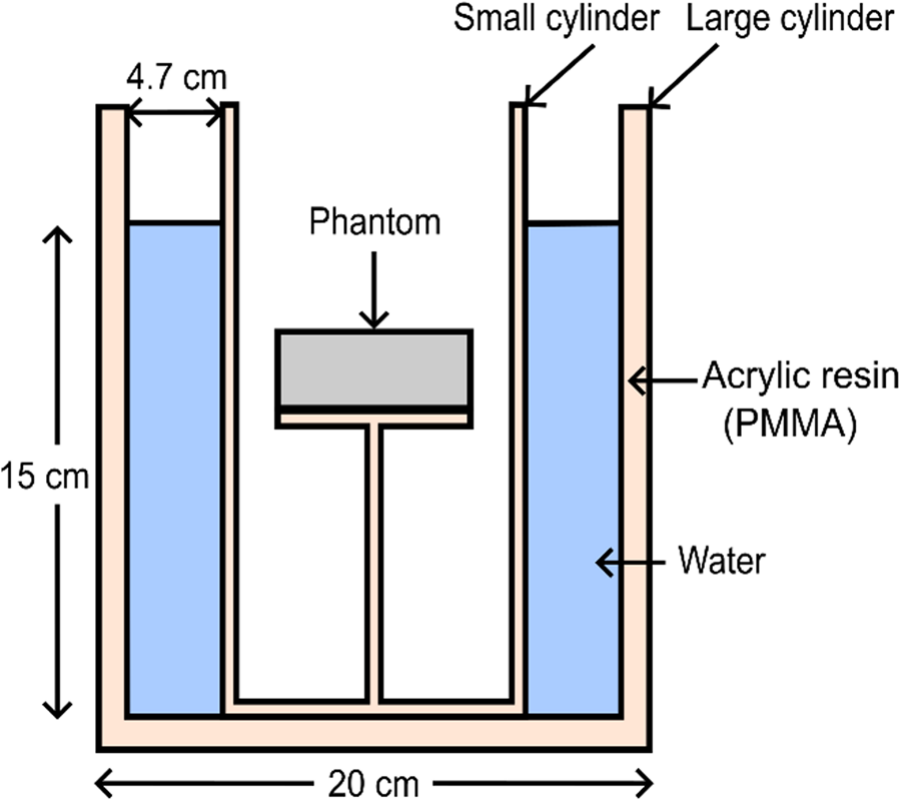
Schematic illustration of the phantom placed in the water tank. A small cylinder was fixed concentrically inside a large cylinder, water was filled in the space between them, and the phantom was positioned at the center of the small cylinder. PMMA: polymethyl methacrylate

To simulate the oral cavity and buccal soft tissues, a double-cylinder water tank structure was fabricated. Both the large cylinder (height, 20 cm; diameter, 20 cm; thickness, 3 mm) and small cylinder (height, 20 cm; diameter, 10 cm; thickness, 2 mm) were made of acrylic resin (polymethyl methacrylate, PMMA). The bottom surface of the small cylinder was fixed to the center of the large cylinder using cyanoacrylate adhesive. The space between the two cylinders was filled with water to simulate buccal soft tissue. The small cylinder simulated the oral cavity. A phantom representing the mandible was placed on a pedestal at the center of the small cylinder. This phantom consisted of acrylic material representing cancellous bone, a PTFE tube representing the mandibular canal, and a titanium screw representing the implant body. The mandibular cortical surface was reproduced using a 0.5 mm-thick aluminum plate (Figs. 1 and 2).

### Imaging modality

CBCT imaging was performed using the 3D Accuitomo 17F (J. Morita Corp., Kyoto, Japan). Image reconstruction was performed by extracting DICOM data using i-Dixel (J. Morita Corp., Kyoto, Japan), and observer measurements were analyzed using ImageJ (version 1.54 g; NIH, Bethesda, MD). Images were reviewed on two liquid crystal display (LCD) monitors: a 23-inch general-purpose LCD monitor (EIZO FlexScan EV2336W-ZBK; EIZO Corp., Ishikawa, Japan) and a 21.2-inch medical-grade LCD monitor (EIZO RadiForce RX340; EIZO Corp., Ishikawa, Japan).

### Determination of actual distance

A thickness gauge (model 65 M; Niigata Seiki, Niigata, Japan) was used to set the fine interspace distances. The gauge was inserted as a spacer to adjust the gap, and the screw was gently rotated by hand until contact was achieved. Using a marking on the screw head as a reference, the screw was rotated 45° counterclockwise to remove the gauge and then tightened again by rotating 45° clockwise, thereby ensuring reproducible gap distances. According to the accuracy specifications of the Japanese Industrial Standards (JIS B 7527), the nominal tolerance of the thickness gauge is 0.01–0.02 mm for thicknesses of 0.1–0.7 mm and ± 0.035 mm for 1.0 mm. To eliminate play between the screw hole and screw, softened paraffin wax was thinly applied for fixation. A level (model INC-RT80; Akatsuki Seisakusho, Kyoto, Japan) with sensitivity 0.25 mm/m was used to confirm horizontality.

To confirm that the titanium screw and the PTFE tube were not in direct contact, a 20 × magnifying loupe (Nikon 8D, Tokyo, Japan) was used. Before placing the phantom, a level placed on the pedestal was used to confirm horizontality each time the imaging conditions were changed, thereby maintaining the pedestal inclination at < 1° (an inclination of 1° can introduce an error of approximately 1.8% relative to the object length).

In this experiment, Distance was defined as the shortest distance from the most protruding part of the titanium screw to the outer surface of the PTFE tube.

### Imaging conditions

**Fig. 3:**
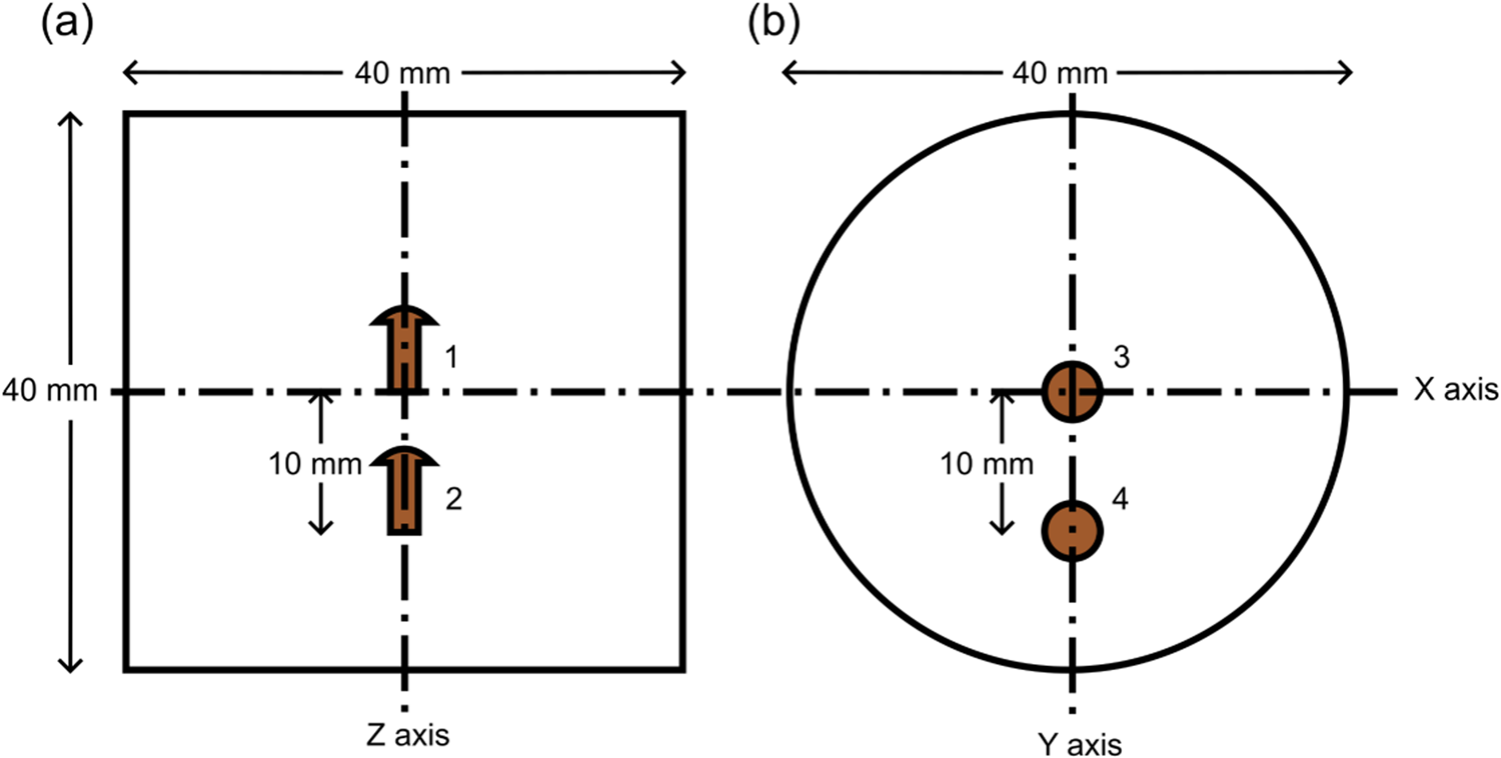
Schematic illustration showing the screw positions used to define the four coordinate combinations. **a** Projection of the screw positions onto the X–Z plane. **b** Projection of the screw positions onto the X–Y plane. The square in (**a**) and circle in (**b**) indicate the radiation field (40 × 40 × 40 mm). The numbers 1–4 denote four possible screw positions. Because (**a**) and (**b**) are orthogonal projections of the same 3D locations, each coordinate condition corresponds to two numbers: XY0Z0 (1 and 3), XY10Z0 (1 and 4), XY0Z10 (2 and 3), and XY10Z10 (2 and 4)

Imaging was performed under the following conditions: exposure time of 30.8 s, scan angle of 360°, Hi-Res mode, field of view (FOV) of 40 × 40 mm, and a voxel size of 0.08 × 0.08 × 0.08 mm. Variable parameters were tube voltage (Voltage) and tube current, set at either 65 kV/3 mA or 80 kV/2 mA. The distance between the titanium screw and the PTFE tube was set at eight levels (0.0, 0.1, 0.2, 0.3, 0.4, 0.5, 0.7, and 1.0 mm). The screw position (Position) within the FOV was set at four locations: at the FOV center (XY0Z0), 10 mm horizontally from the center (XY10Z0), 10 mm vertically from the center (XY0Z10), and 10 mm both horizontally and vertically from the center (XY10Z10) (Fig. 3). This resulted in a total of 64 experimental conditions. Each condition was imaged three times, yielding a total of 192 images.

### Evaluators and measurement method

Distance measurements were performed by dentists involved in implant treatment using predefined images.

The evaluations were conducted by five observers: three board-certified oral and maxillofacial radiologists and two dentists engaged in prosthodontic treatment. Each observer measured all 192 images in a randomized order using both a medical-grade monitor (used in radiology departments) and a general-purpose monitor (used in other general clinical departments).

For measurement, the following single instruction was provided:

“Measure the shortest distance between the titanium screw and the PTFE tube, that is, the shortest distance from the most protruding part of the titanium screw to the closest point on the outer surface of the PTFE tube.”

Measurements were performed under room lighting conditions similar to those used in clinical practice, and adjustment of image settings (window level, window width, and magnification) was left to the discretion of each observer. Measurements on each monitor were completed on the same day, with appropriate breaks, and required approximately 1 h per monitor.

### Statistical analysis

Statistical analyses were performed using R (version 4.4.2; R Foundation for Statistical Computing, Vienna, Austria), with the significance level set at *p* < 0.05. Variability was assessed using the interquartile range (IQR). The true value was defined as the thickness gauge reading. Measurement error (Difference) was calculated as the true value minus the measurement; positive values indicated underestimation and negative values indicated overestimation.

Linear relationships between Distance and the measurements were assessed using correlation coefficients. Linear regression analysis was performed, with the regression coefficients (intercept and slope) reported with 95% confidence intervals (CIs). For subsequent analyses, the absolute value of measurement error (Difference) was used as the dependent variable.

Differences among Positions were evaluated using the Kruskal–Wallis test, and when significant, post-hoc multiple comparisons were performed using Dunn’s test with Holm adjustment. Differences between Voltages (65 and 80 kV) were assessed using the Wilcoxon rank-sum test.

A factorial analysis of variance (ANOVA) was performed with the absolute value of the measurement error (Difference) as the dependent variable and Monitor (monitor type: general-purpose vs. medical-grade; two levels), Position (four levels), Voltage (two levels), and Distance (eight levels) as fixed factors. Homogeneity of variance was assessed using Levene’s test; when violated, an aligned rank transform [16] was applied prior to ANOVA. Post-hoc pairwise comparisons were conducted using estimated marginal means, with Tukey adjustment applied for factors with more than two levels. Where a conventional ANOVA model was used, Tukey’s honestly significant difference (HSD) test was applied.

## Results

### Measurement data

**Fig. 4:**
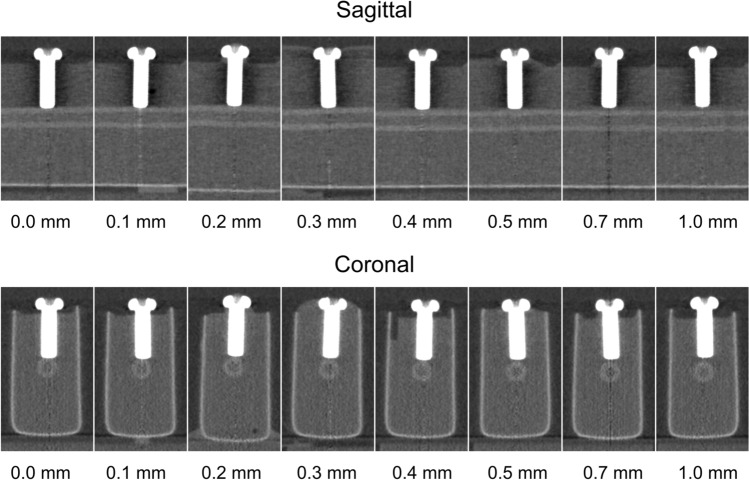
Sagittal and coronal CBCT images at each screw–tube distance. Images were uniformly cropped to a consistent region of interest without rotation or nonlinear manipulation. CBCT: cone-beam computed tomography

**Fig. 5:**
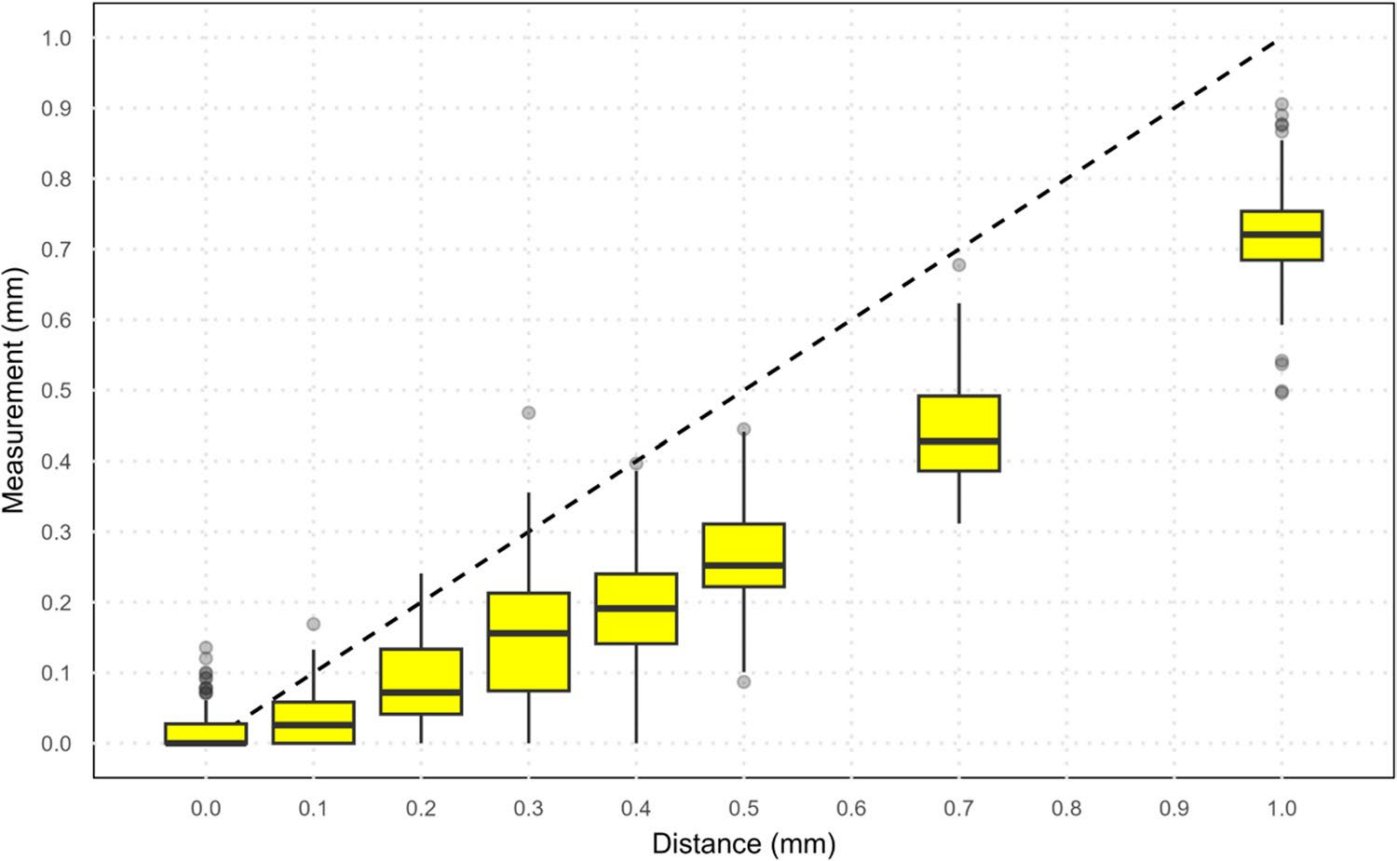
Box plots showing measurements at each true distance. Box plots show the distribution of measurements at each distance. Boxes represent the IQR, horizontal lines indicate the median, and whiskers represent 1.5 × IQR. Outliers are shown as individual points. Each distance group contained n = 80 measurements. The dashed line indicates *y* = *x*. IQR: interquartile range

Figure 4 shows a representative image acquired at Position XY0Z0 with a Voltage of 80 kV and tube current of 2 mA. Figure 5 illustrates the relationship between Distance and the measurements. Because Distance was defined as the shortest distance between two points, it could not take negative values; therefore, measurements at Distance 0.0 mm were excluded from the regression analysis. In addition, because two distinct regression patterns were noted (Fig. 5), analyses were performed separately for the Distance 0.1–0.4 mm group and the Distance 0.5–1.0 mm group. Among the evaluated distances, the largest variability in measurements was observed at a titanium screw–PTFE tube distance of 0.3 mm (IQR = 0.121). In the Distance 0.1–0.4 mm group, the measurements tended to increase with increasing Distance (*r* = 0.995*, p* = 0.005; regression slope = 0.543, 95% CI 0.382–0.704). In contrast, in the Distance 0.5–1.0 mm group, the measurements were approximately proportional to the true values; however, the measurements were consistently smaller than the true values (*r* = 0.999*, p* = 0.011; regression slope = 0.920, 95% CI 0.726–1.115; intercept =  − 0.199, 95% CI  − 0.348 to − 0.051). Inter-rater agreement was also assessed using the intraclass correlation coefficient (ICC). The ICC(2,5) was 0.80 (95% CI 0.65–0.88) for the Distance 0.1–0.4 mm group and 0.97 (95% CI 0.93–0.98) for the Distance 0.5–1.0 mm group.

### Measurement error (Difference)

**Fig. 6:**
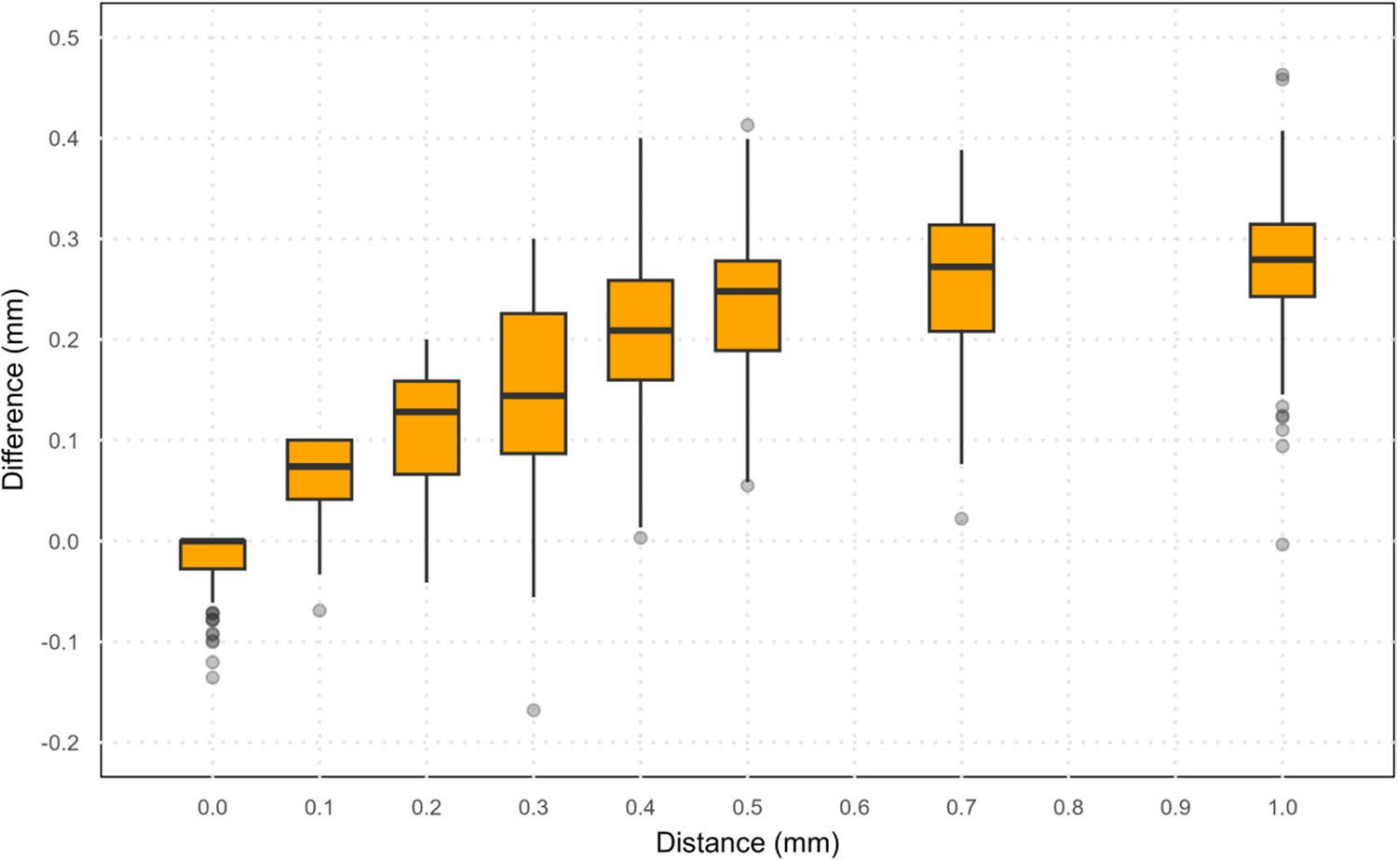
Box plots showing differences between true values and measurements at each distance. Box plots show the distribution of differences, defined as the true value minus measurement (i.e., signed differences), at each distance. Boxes represent the IQR, horizontal lines indicate the median, and whiskers represent 1.5 × IQR. Outliers are shown as individual points. Each distance group contained n = 80 measurements. IQR: interquartile range

Figure 6 shows the relationship between Distance and Difference.

In the Distance 0.1–0.4 mm group, Difference tended to increase with increasing Distance (*r* = 0.993*, p* = 0.007; regression slope = 0.457, 95% CI 0.296–0.618). In contrast, in the Distance 0.5–1.0 mm group, changes in Difference with Distance were small, and the measurements were smaller than the true values by an approximately constant amount (*r* = 0.982*, p* = 0.121; regression slope = 0.080, 95% CI  − 0.115 to 0.274; intercept = 0.199, 95% CI 0.051–0.348).

Subsequently, the absolute value of Difference was used to examine the effects of each factor and their interactions. In this analysis, because the absolute value of Difference was always positive, all data, including those at Distance 0.0 mm, were included.

**Fig. 7:**
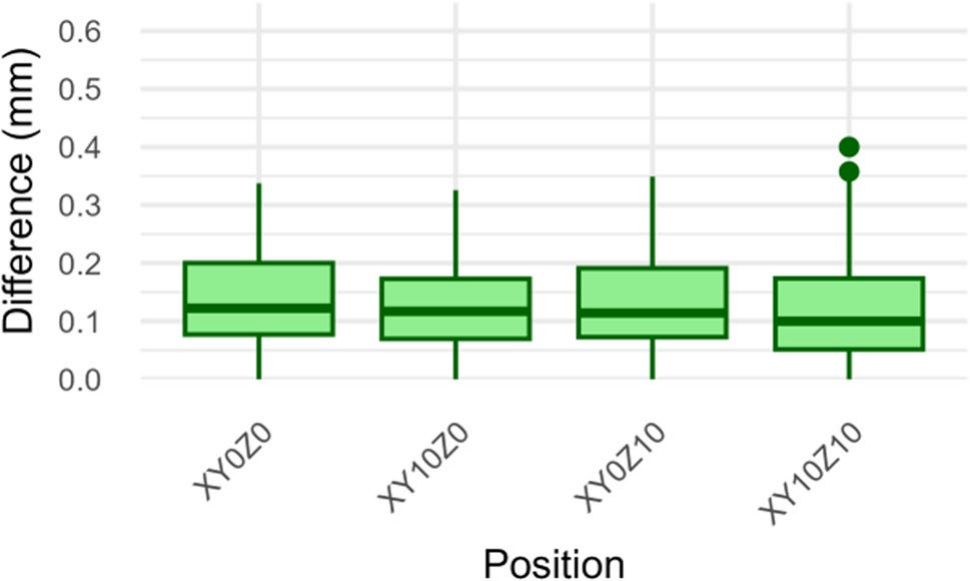
Box plots showing absolute differences among positions (0.0–0.4 mm). Box plots show the distribution of absolute differences, defined as the absolute value of the true value minus the measurement (true value − measurement) for each position within the 0.0–0.4 mm distance range. Boxes represent the IQR, horizontal lines indicate the median, and whiskers represent 1.5 × IQR. Outliers are shown as individual points. Each position group contained n = 100 measurements. IQR: interquartile range

**Fig. 8:**
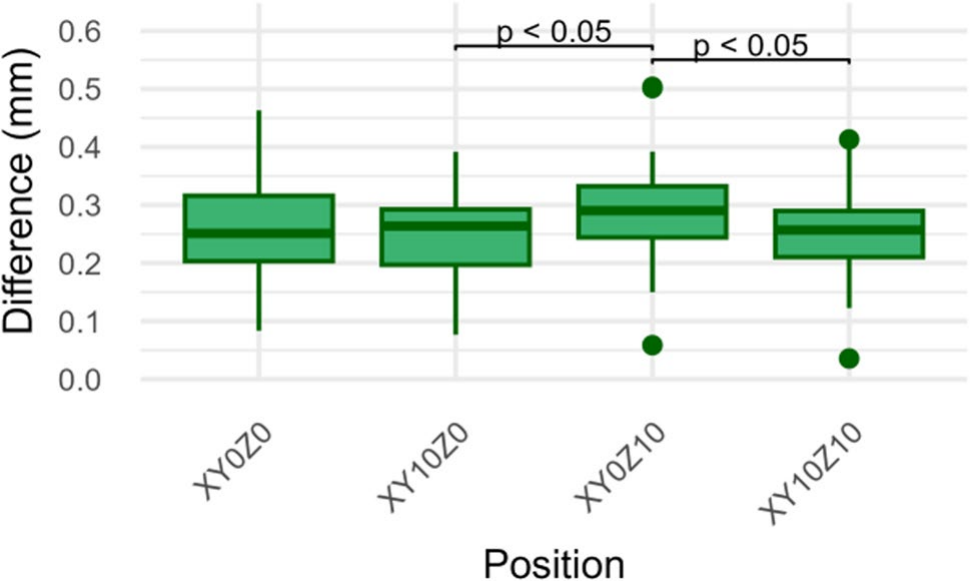
Box plots showing absolute differences among positions (0.5–1.0 mm). Box plots show the distribution of absolute differences, defined as the absolute value of the true value minus the measurement (true value − measurement) for each position within the 0.5–1.0 mm distance range. Boxes represent the IQR, horizontal lines indicate the median, and whiskers represent 1.5 × IQR. Outliers are shown as individual points. Statistical differences among positions were analyzed using the Kruskal–Wallis test followed by pairwise comparisons with Dunn’s test with Holm adjustment. *p* < 0.05 was considered statistically significant. Each position group contained n = 60 measurements. IQR: interquartile range

With respect to changes in Position, no significant differences were observed in the Distance 0.0–0.4 mm group (*p* = 0.354, Fig. 7). In contrast, significant differences were observed in the Distance 0.5–1.0 mm group (*p* = 0.019). Post-hoc multiple comparisons using Dunn’s test with Holm adjustment showed that absolute Differences were significantly smaller at Positions XY10Z0 and XY10Z10 than at Positions XY0Z10 (XY10Z0 vs. XY0Z10, *p* = 0.049; XY10Z10 vs. XY0Z10, *p* = 0.046; Fig. 8).

**Fig. 9:**
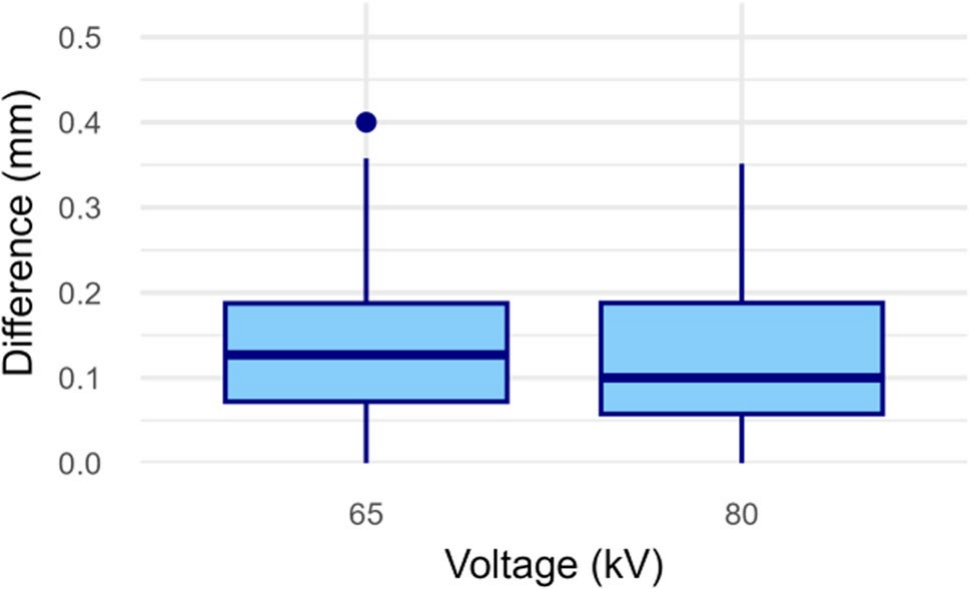
Box plots showing absolute differences between voltages (0.0–0.4 mm). Box plots show the distribution of absolute differences, defined as the absolute value of the true value minus the measurement (true value − measurement) for each voltage (65 kV and 80 kV) within the 0.0–0.4 mm distance range. Boxes represent the IQR, horizontal lines indicate the median, and whiskers represent 1.5 × IQR. Outliers are shown as individual points. Each voltage group contained n = 200 measurements. IQR: interquartile range

**Fig. 10:**
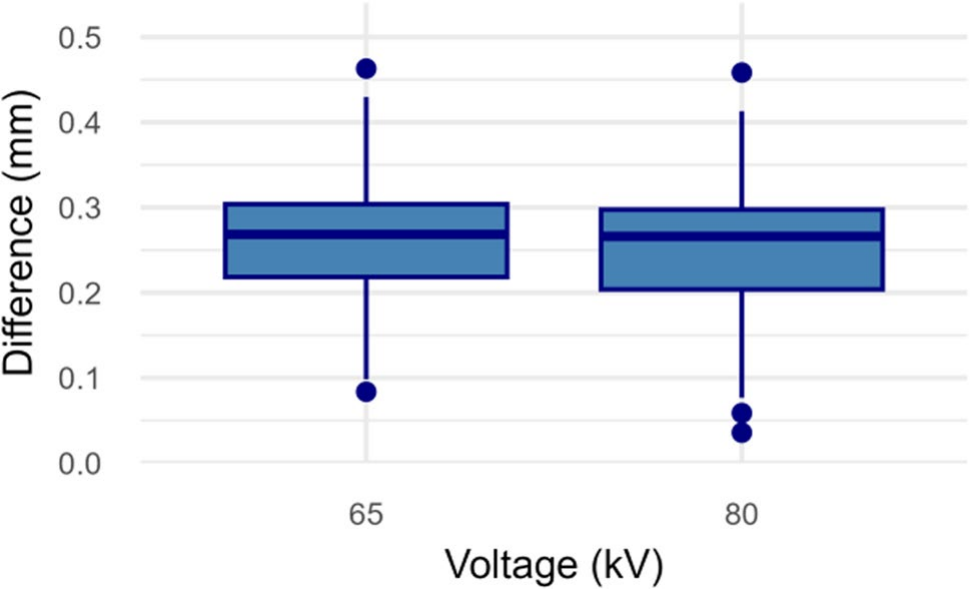
Box plots showing absolute differences between voltages (0.5–1.0 mm). Box plots show the distribution of absolute differences, defined as the absolute value of the true value minus the measurement (true value − measurement) for each voltage (65 kV and 80 kV) within the 0.5–1.0 mm distance range. Boxes represent the IQR, horizontal lines indicate the median, and whiskers represent 1.5 × IQR. Outliers are shown as individual points. Each voltage group contained n = 120 measurements. IQR: interquartile range

Regarding Voltage, no significant differences were observed between 65 and 80 kV in either the Distance 0.0–0.4 mm group (*p* = 0.149, Fig. 9) or Distance 0.5–1.0 mm group (*p* = 0.283, Fig. 10).

### Factorial analysis

In the Distance 0.0–0.4 mm group, no significant differences were observed for Monitor or Position, whereas significant effects were found for Voltage (*p* = 0.004) and Distance (*p* < 0.001). In addition, significant interactions were observed for Position × Distance (*p* = 0.020) and Position × Voltage (*p* = 0.016). Post-hoc pairwise comparisons for Distance based on estimated marginal means with Tukey adjustment showed that Difference increased stepwise with increasing Distance (*p* < 0.001; Table 1).

**Table 1:** ANOVA^†^ and multiple comparison results for absolute differences^‡^ in the 0.0–0.4 mm range

Term	Df	F	*p*	Multiple comparisons	*p*
Monitor	1	0.028	0.867		
Position	3	2.380	0.070		
Voltage	1	8.411	0.004**	65 kV > 80 kV	0.004*
Distance	4	184.102	< 0.001***	< 0.1 < 0.2 < 0.3 < 0.4 mm	< 0.001***
Position × distance	12	2.053	0.020*		
Position × voltage	3	3.487	0.016*		

^†^: aligned rank transform was applied prior to ANOVA [16]. ^‡^: absolute differences were calculated as the true value minus the measurement, expressed as a positive value. ANOVA: analysis of variance; Df: degrees of freedom; F: F value; *p*: probability value. Post-hoc pairwise comparisons were conducted using estimated marginal means (emmeans). Tukey adjustment was applied where appropriate for factors with more than two levels. Among the multiple comparisons for Distance, the smallest difference was observed between 0.2 and 0.3 mm (*p* = 0.008), whereas all other pairs showed *p* < 0.001. * *p* < 0.05, ** *p* < 0.01, *** *p* < 0.001

In the Distance 0.5–1.0 mm group, no significant differences were observed for Monitor or Voltage, whereas significant effects were found for Position (*p* = 0.015) and Distance (*p* = 0.001). Significant interactions were also observed for Position × Distance (*p* = 0.037) and Position × Voltage (*p* = 0.014). Tukey’s multiple comparisons for Distance showed that Difference at Distance 1.0 mm was significantly greater than that at 0.5 mm (*p* = 0.001; Table 2).

**Table 2:** ANOVA and multiple comparison results for absolute differences^†^ in the 0.5–1.0 mm range

Term	Df	F	*p*	Multiple comparisons	*p*
Monitor	1	0.008	0.930		
Position	3	3.655	0.015**	XY10Z0 < XY0Z10 XY10Z10 < XY0Z10	0.018* 0.042*
Voltage	1	2.632	0.110		
Distance	2	7.012	0.001**	0.5 < 1.0 mm	0.001***
Position × distance	6	2.289	0.037*		
Position × voltage	3	3.652	0.014*		

^†^: absolute differences were calculated as the true value minus the measurement, expressed as a positive value. ANOVA: analysis of variance; Df: degrees of freedom; F: F value; *p*: probability value. Post-hoc pairwise comparisons were conducted using Tukey’s honestly significant difference (HSD) test. * *p* < 0.05, ** *p* < 0.01,*** *p* < 0.001

## Discussion

Based on the ANOVA results of the present study, the distance between the titanium screw and the PTFE tube had the greatest influence on measurement error compared with monitor type, measurement position, and tube voltage (Table 1). Notably, measurement instability was most pronounced at shorter distances, particularly around 0.3 mm under the present experimental settings (Figs. 5 and 6).

Previous studies have shown that, in the vicinity of metal objects, beam hardening, scattered radiation, partial volume effects, and exponential edge-gradient effect occur simultaneously, causing metal boundaries to appear expanded outward relative to their actual positions, referred to as the blooming artifact. Moreover, grayscale fluctuations at metal boundaries are large, making these regions the most unstable [17]. In the present study, the distance range exhibiting the greatest measurement error and variability likely corresponded to the artifact expansion zone around metal (approximately 0.2–0.4 mm), where the titanium screw tip coincided with the most unstable image reconstruction region.

Against this background, in the Distance 0.0–0.4 mm group, although a strong association between titanium screw–PTFE tube distance and measurement error was observed, the magnitude of the error was on the order of the system voxel size (0.08 mm^3^). The lower ICC(2,5) observed in the 0.1–0.4 mm group indicates that measurements taken at shorter distances were more prone to inter-rater variability, although the overall level of agreement remained within acceptable limits. The measurements were unstable, resulting in insufficient accuracy. Even a small difference of 0.1 mm produced substantial variability in measurements, indicating that accurate distance assessment using CBCT is difficult in this distance range.

In contrast, in the Distance 0.5–1.0 mm group, a strong linear relationship with a slope close to unity was observed between the true values and the measurements, indicating high reproducibility. However, the measurements did not coincide with the true values; instead, they were consistently underestimated by 0.2–0.3 mm. Accordingly, when a distance of ≥ 0.5 mm is observed on CBCT, the actual anatomical distance should be interpreted as being approximately 0.2–0.3 mm greater than the measurement.

From an MTF-based perspective, measurement instability was pronounced up to approximately 0.4 mm [18], whereas a tendency for the measurements to stabilize was observed around 0.5 mm. This finding is consistent with a previous report suggesting that CBCT may be unreliable for discriminating distances ≤ 0.4 mm. Based on this characteristic, if the resolution of a gap can be confirmed using a phantom in which the titanium screw–PTFE tube distance is fixed at 0.5 mm, such a phantom may be useful for quality assurance testing and may serve as an indicator that sufficient image quality has been achieved.

Clinically, although strict distance assessment is desired when an implant is within 1.0 mm of the mandibular canal, there is limited evidence that current CBCT systems can guarantee true measurement accuracy at the 0.1-mm level. A key contribution of the present study is that it quantitatively demonstrated, for the first time, how a 0.1-mm difference in distance in the submillimeter range is depicted and manifests as a measurement error under conditions simulating the presence of an implant body.

Furthermore, when the distance between the implant apex and the superior wall of the mandibular canal is judged to be ≥ 0.5 mm on CBCT images, the actual distance is likely to be greater, suggesting a lower clinical risk. Owing to the scattered radiation from the implant, the implant apex and the superior wall of the mandibular canal may appear to be in contact on CBCT images even when an actual separation of approximately 0.2 mm exists. Such misinterpretation may lead to unnecessary implant removal.

In recent years, dental CBCT and simulation software have become essential for implant planning [19]. However, due to multiple factors, such as beam hardening, partial volume effects, and limited reproducibility of mandibular canal tracing, distances measured on images do not necessarily correspond to true anatomical distances. Previous studies have reported an association between implant–canal proximity and the occurrence of neurosensory disturbances [10]. Although these findings are based on MDCT research, a minimum distance of at least 1.5 mm has been clinically recommended when occlusal force and prosthetic conditions are considered [11]. The present findings also suggest that when the distance measured on CBCT images is approximately 1.5 mm, the actual distance may be approximately 1.7–1.8 mm. Therefore, if a safety margin of 1.5 mm is visually confirmed in planning software, complications such as nerve injury may be less likely. Nevertheless, it is important to recognize that distances displayed on images do not accurately reflect true anatomical distances.

Regarding Position, outliers were observed at XY10Z10, indicating that when the distance between the implant apex and the superior wall of the mandibular canal is very small (≤ 0.4 mm), placement far from the center of the radiation field in both the horizontal and vertical directions should be avoided (Fig. 7). Moreover, even at greater distances, outliers were observed at XY0Z10 and XY10Z10; therefore, positioning the target on a plane passing through the center of the radiation field is recommended. Lagravère et al. reported that the CBCT coordinate system is highly sensitive to positional changes along the Z-axis, and that even a small shift of 0.25–1.0 mm at a reference point may be amplified into errors of 1 mm to several mm at other landmarks and up to ± 10 mm at distal sites [20]. Ozaki et al. also reported that CBCT spatial resolution is highest at the radiation center and center of the FOV, and decreases in a position-dependent manner toward the periphery, indicating that the effective resolution of CBCT varies with acquisition conditions and object position [18].

Regarding tube voltage, at distances ≤ 0.4 mm between the titanium screw and the PTFE tube, measurement error was smaller at 80 kV than at 65 kV; however, no significant differences were observed at distances ≥ 0.5 mm (Figs. 9 and 10). Overall, no significant effect of tube voltage was detected in this study, suggesting that tube voltage is not a consistent determinant of measurement accuracy. This finding aligns with previous reports that variations in tube voltage have a limited influence on image characteristics and metal artifacts in CBCT [21]. At the tube voltage generally used in implant CBCT (80–95 kV) [22], the effect of beam hardening is often more pronounced than that of scattered radiation. Because titanium strongly absorbs low-energy components, lower tube voltages enhance beam hardening and increase artifacts [23]. Therefore, higher tube voltages within the allowable range of the device should be selected when evaluating the distance between an implant and the mandibular canal. A previous study reported that metal artifact reduction (MAR), a reconstruction algorithm used to suppress metal-induced artifacts in CBCT images, can reduce diagnostic performance in assessing contact between an implant and the mandibular canal and may cause these structures to appear in contact even when a gap is present [24]. From this perspective, disabling MAR may be preferable when the objective is precise distance evaluation.

In addition, no significant difference in measurement accuracy was observed between the general-purpose monitor used in general dental practice and the dedicated diagnostic monitor used in the radiology department. This result suggests that distance measurements obtained in a typical dental office environment do not differ substantially from those obtained in specialized reading environments such as university hospitals.

Overall, although tube voltage and monitor conditions can potentially influence measurements, their effects are smaller than that of the distance itself and may vary depending on the device and acquisition conditions. Therefore, rather than optimizing a single parameter, distance evaluation should be performed with a comprehensive understanding of the imaging conditions. In addition to visual assessment on routine monitors, objective indices such as plot profiles and full width at half maximum may provide supplementary information for evaluating boundary depiction around the implant.

## Limitations

This study has a few limitations. The number of observers was small, comprising three oral and maxillofacial radiologists and two prosthodontists; therefore, evaluation of interobserver variability was limited. Future studies should include a larger number of observers to allow more detailed assessments.

Further studies under conditions that more closely reflect clinical situations are also warranted. Specifically, evaluation using phantoms incorporating M6 titanium screws, multiple implants, or simulated dental arches extending beyond the FOV will enable assessment of measurement accuracy across a wider range of imaging scenarios, and such studies are planned for the future.

## Conclusions

Clinically, although the present findings were obtained under experimental conditions, they suggest that CBCT-based evaluation of the distance between an implant body and the mandibular canal in the submillimeter range may have inherent limitations. Distances of ≤ 0.4 mm may not be reliably distinguished, and even when CBCT indicates a clearance of ≥ 0.5 mm, the true distance may be approximately 0.2–0.3 mm greater due to systematic underestimation. Consequently, CBCT-derived measurements should be interpreted with caution when assessing proximity to the mandibular canal, and this bias should be taken into account when determining safety margins in implant planning and postoperative evaluation.

## Data Availability

The datasets generated and/or analyzed during the current study are available from the corresponding author on reasonable request.

## Abbreviations

ANOVA: Analysis of variance
CBCT: Cone-beam computed tomography
CI: Confidence interval
FOV: Field of view
HSD: Honestly significant difference
ICC: Intraclass correlation coefficient
IQR: Interquartile range
LCD: Liquid crystal display
MAR: Metal artifact reduction
MDCT: Multi-detector row CT
MTF: Modulation transfer function
PMMA: Polymethyl methacrylate
PTFE: Polytetrafluoroethylene
